# Effect of borax on immune cell proliferation and sister chromatid exchange in human chromosomes

**DOI:** 10.1186/1745-6673-4-27

**Published:** 2009-10-30

**Authors:** Malinee Pongsavee

**Affiliations:** 1 Department of Medical Technology, Faculty of Allied Health Sciences, Thammasat University, Rangsit Campus, Patumthani 12121, Thailand

## Abstract

**Background:**

Borax is used as a food additive. It becomes toxic when accumulated in the body. It causes vomiting, fatigue and renal failure.

**Methods:**

The heparinized blood samples from 40 healthy men were studied for the impact of borax toxicity on immune cell proliferation (lymphocyte proliferation) and sister chromatid exchange in human chromosomes. The MTT assay and Sister Chromatid Exchange (SCE) technic were used in this experiment with the borax concentrations of 0.1, 0.15, 0.2, 0.3 and 0.6 mg/ml.

**Results:**

It showed that the immune cell proliferation (lymphocyte proliferation) was decreased when the concentrations of borax increased. The borax concentration of 0.6 mg/ml had the most effectiveness to the lymphocyte proliferation and had the highest cytotoxicity index (CI). The borax concentrations of 0.15, 0.2, 0.3 and 0.6 mg/ml significantly induced sister chromatid exchange in human chromosomes (*P *< 0.05).

**Conclusion:**

Borax had effects on immune cell proliferation (lymphocyte proliferation) and induced sister chromatid exchange in human chromosomes. Toxicity of borax may lead to cellular toxicity and genetic defect in human.

## Background

Borax (Na_2_B_4_O_2_(H_2_O)_10_) is a low toxicity mineral with insecticidal, fungicidal and herbicidal properties. The basic structure of borax contains chains of interlocking BO_2_(OH) triangles and BO_3_(OH) tetrahedrons bonded to chains of sodium and water octahedrons. Borax occurs naturally in evaporate deposits produced by the repeated evaporation of seasonal lakes. It is a precursor for sodium perborate monohydrate that is used in detergents. Borax can be produced synthetically from other boron compounds. It is usually a white powder consisting of soft colorless crystals that dissolve easily in water. Borax is used in detergents and cosmetics, as an ingredient in enamel glazes, glass, pottery, and ceramics, to make buffer solutions. It is used mixed with water as a flux when soldering jewelry metals such as god or silver [1].

Borax has the toxicity to humans, including reproductive and developmental toxicity, neurotoxicity, and nephrotoxicity. The degree of borax toxicity depends on the dose or concentration that the human received. The most sensitive endpoints of borax toxicity is developmental and reproductive toxicity [2]. Borax causes irritation of skin and respiratory tract. The gastrointestinal tract, skin, vascular system and brain are the principal organs and tissues affected. It causes nausea, persistant vomiting, abdominal pain, diarrhea, erythematous and exfoliative rash, unconsciousness, depression and renal failure. Brockman et al., 1985 reported about borax toxicity in animals. Chunks of borax were used to control in the pH^+ ^in drilling muds. Cattle consumed these chunks. They appeared depressed, were dehydrated and some had diarrhea. In the dead animals, the predominant lesion was hemorrhagic gastroenterititis [3].

Food additives are substances added to food to preserve flavor or improve its taste and appearance. Food additives are one of the causes in many cancer types [4]. Carrageenan is one of the food additive that involve in cancer. It is a naturally occurring gum derived from red seaweed. It is associated with loss of mammary myoepithelial cells in tissue culture provides a potential mechanism for increasing invasive mammary carcinoma [5] and induced colonic neoplasia in animal models [6]. Borax is used as a food additive in some countries with the E number *E285*. Although borax has reproductive toxicity, nephrotoxicity and neurotoxicity in human but the borax toxicity about human genetic materials and humoral immune cell still need to be investigated for food additive carcinogenesis. The defect in genetic material and immune cell development involves in human carcinogenesis. In this study, we studied about the toxic effects of borax on immune cell proliferation (lymphocyte proliferation) and genotoxicity (sister chromatid exchange). We studied the toxic effect on immune cell proliferation by MTT assay and genotoxicity (cytogenetic level) by Sister Chromatid Exchange technic (SCE technic).

MTT [3-(4,5-dimethyl-2-thiazolyl)-2,5-diphenyl-2H tetrazolium bromide] colorimetric assay is a standard colorimetric assay for measuring cellular growth. It has been developed by Mosmann. It can also be used to determine cytotoxicity of toxic materials. Yellow MTT is reduced to purple formazan in the mitochondria of living cells. A solubilization solution is added to dissolve the insoluble purple formazan product into a colored solution. The absorbance of this colored solution can be quantified by measuring at a certain wavelength (usually between 500 and 600 nm.) by a spectrophotometer. The absorption maximum is dependent on the solvent employed. This reduction takes place only when mitochondrial reductase enzymes are active, and therefore conversion can be directly related to the number of living cells. When the amount of purple formazan produced by cells treated with an agent is compared with the amount of formazan produced by untreated control cells, the effectiveness of the agent in causing death of cells can be deduced, through the production of a dose-response curve [7]. Various technical modifications for the MTT assay on different cell lines have done in many experiments about cancer. The MTT assay can be used for the chemosensitivity testing of short-term cell lines derived from human brain tumors [8].

Sister chromatid exchange (SCEs) represent the interchange of DNA replication products at apparently homologous chromosomal loci. These exchanges involve DNA breakage and reunion. Sister Chromatid Exchange technic (SCE technic) affords the opportunity for cytological detection of DNA interchange. This technic is used as a sensitive means of monitoring DNA damage. It is useful for assessing the cytogenic impact of clastogenic agents on chromosomes. It can be performed in cultured cells (in vitro) or on cells from intact animals given BrdU (in vivo). Many agents found to induce SCEs are well-known mutagens and/or carcinogens [9]. The increased resolution of SCE detection afforded by fluorescence or Giemsa technic has permitted localization of SCEs relative to chromosome-banding patterns. In human chromosomes, SCEs occur preferentially in Q-negative bands or at the junction of Q-positive and Q-negative regions [10]. The toxic effects of borax to immune cell proliferation and genotoxicity in human were studied (in vitro) to verify borax toxicity.

## Methods

### Blood samples

Heparinized blood from 40 consenting healthy male were collected. The participants were given consent to donate blood for study in the borax toxicity research. This study was approved by Thammasat University ethic committee.

### Studying the effect of borax on immune cell proliferation (lymphocyte proliferation) by MTT assay

Forty human lymphocyte cultures of forty heparinized blood samples were cultured in RPMI medium (Gibco BRL Life Technologies, San Diego, CA, USA) plus 10% fetal calf serum (Invitrogen, Carlsbad, CA, USA) and penicillin-streptomycin antibiotic. Phytohemagglutinin was added in RPMI medium as the mitogen and incubated for 72 hours. After 72 hours incubation, a total of 1 × 10^5 ^cells/well from each lymphocyte culture were selected in the 24-well plate and incubated for 24 hours.

The borax concentrations of 0.1, 0.15, 0.2, 0.3 and 0.6 mg/ml were tested in this study. These doses were high as compared to being used as food additive but it is likely that due to high consumption it may be accumulative. Forty lymphocyte cultures from forty blood samples were tested in each borax concentration. After 24 hours incubation, various concentrations of borax added to the wells to get the final concentration of 0.1, 0.15, 0.2, 0.3 and 0.6 mg/ml. The cells were incubated for an additional 24 hours. After 24 hours, 0.5 ml of 300 μg/ml MTT in phosphate buffer saline (PBS) were added to each well and incubated for 4 hours at 37°C. The medium was removed and formazan was dissolved in DMSO and the optical density was measured at 570 nm. using a Bio-assay reader. MTT assay of forty lymphocyte cultures in each borax concentration was carried out in duplicate fashion. Mean absorbance was calculate for the control wells and for each borax concentration in the test wells. The degree of immune cell proliferation (lymphocyte proliferation) sensitivity to borax toxicity was based on the cytotoxicity index (CI). The percent of cytotoxicity index (%CI = [1-OD_570 _treated/OD_570 _control] × 100) was calculated for each borax concentration [11].

### Studying the effect of borax on genotoxicity (sister chromatid exchange in human chromosomes) by Sister Chromatid Exchange technic (SCE technic)

Forty human lymphocyte cultures of forty heparinized blood samples were cultured in RPMI medium (Gibco BRL Life Technologies, San Diego, CA, USA) plus 10% fetal calf serum (Invitrogen, Carlsbad, CA, USA) and penicillin-streptomycin antibiotic. Phytohemagglutinin was added in RPMI medium as the mitogen. One ml of 6 μg/ml BrdU was added in each lymphocyte culture. The borax solutions, 0.1, 0.15, 0.2, 0.3 and 0.6 mg/ml were added in forty lymphocyte cultures respectively. Forty lymphocyte cultures from forty samples were tested in each borax concentration. After 72 hours incubation, colcemid solution was added 30 minutes prior to the harvest. Metaphase cells were harvested by centrifugation, treated with 0.075 M KCl and fixed in methanol: acetic (3:1). Slides were prepared and stained by the fluorescence plus Giemsa technic [12]. SCEs from forty lymphocyte cultures in each borax concentration were detected. SCEs were observed under microscope and scored in forty five metaphases per sample [13].

### Statistical analysis

Statistical analysis for study the toxicity of each borax concentration on immune cell proliferation (lymphocyte proliferation) by MTT assay and study about the genotoxicity effect were done by ANOVA test.

## Results

### Studying the effect of borax on immune cell proliferation (lymphocyte proliferation)

**Figure 1:**
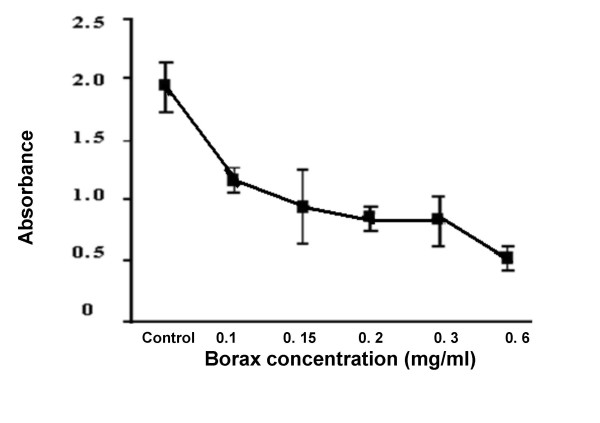
**The correlation between measured absorbance (mean ± SD) and the various borax concentrations by MTT assay**. Mean absorbance related to the numbers of living lymphocytes. The lymphocyte proliferation was decreased when the borax concentration increased. Borax effects on immune cell proliferation (lymphocyte proliferation).

**Figure 2:**
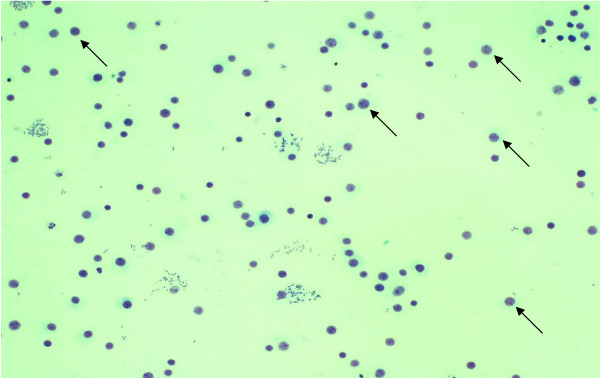
**Lymphocyte proliferation in the control group**. The black arrows indicated many living lymphocytes in this group.

**Figure 3:**
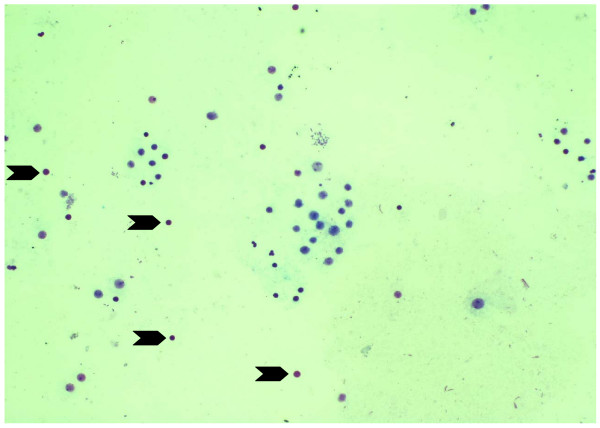
**Lymphocyte proliferation in the 0.2 mg/ml borax concentration experimental subgroup**. The black arrows indicated the dead lymphocytes in this subgroup. Borax effects on lymphocyte proliferation.

**Figure 4:**
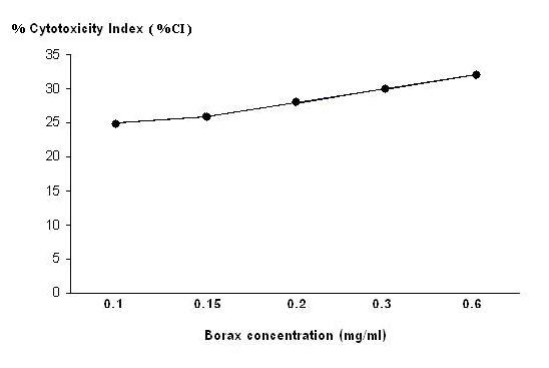
**The correlation between the cytotoxicity index (CI) and the borax concentrations**. It showed that the borax concentration of 0.6 mg/ml had the most cytotoxic effectiveness on immune cell proliferation (lymphocyte proliferation).

The cultures of human lymphocyte were treated with borax at final concentration of 0.1, 0.15, 0.2, 0.3 and 0.6 mg/ml for 24 hours. The value of mean absorbance reflected the anti-proliferation of lymphocyte by borax. Mean absorbance conversion can be directly related to the number of living lymphocytes. The results indicated that at the borax concentrations of 0.15, 0.2, 0.3 and 0.6 mg/ml, lymphocytes showed low proliferation as compared to that of control group and 0.1 mg/ml borax concentration (Figure 1). The numbers of living lymphocytes were decreased when the borax concentrations increased (Figures 2, 3). The proliferation of lymphocytes was inhibited by borax. The 0.15 mg/ml borax concentration was the minimum borax concentration which was toxic for immune cell proliferation (lymphocyte proliferation). The different borax concentrations for standardization (0.1 - 0.6 mg/ml) were used in this study. Any doses between 0.1 mg/ml to 0.6 mg/ml borax concentrations gave comparable results and the cytotoxicity index (CI) was calculated for each borax concentration. The borax concentration of 0.6 mg/ml had the most effectiveness to the lymphocyte proliferation and had the highest cytotoxicity index (CI) in this study (Figure 4). The 50% inhibitory concentration (IC_50_) was 0.9 mg/ml (unpublished data). The results indicated the correlation between the immune cell proliferation and the borax cytotoxicity.

### Studying the effect of borax on genotoxicity (sister chromatid exchange in human chromosomes)

**Figure 5:**
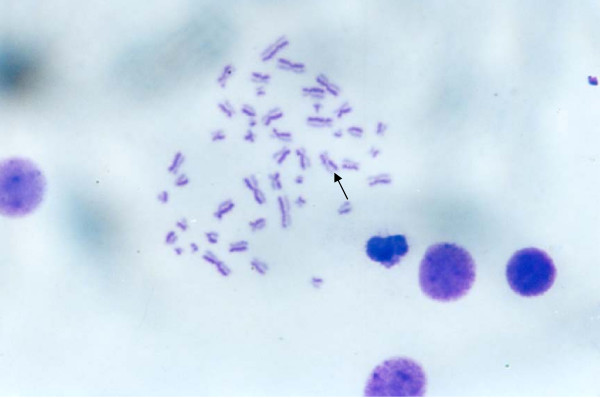
**SCE in the control group**. The black arrow indicated SCE in human chromosome.

**Figure 6:**
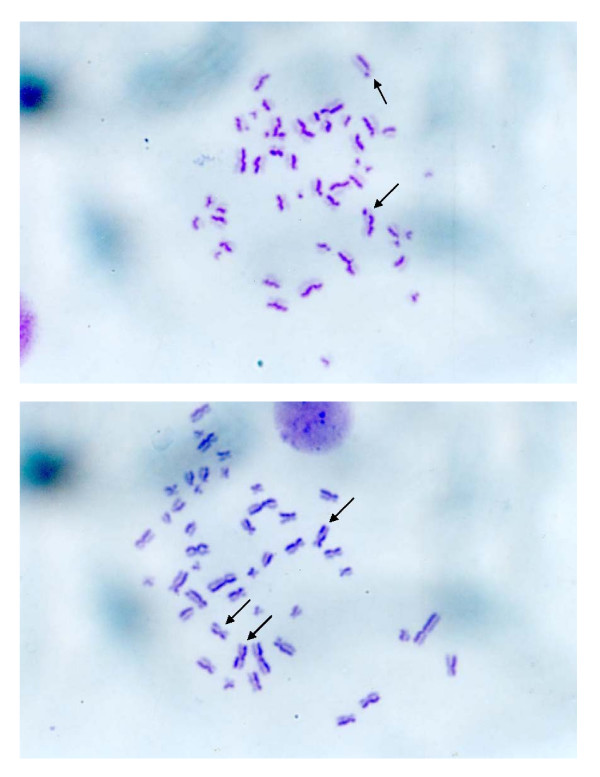
**SCE frequencies in the 0.15 mg/ml (6A), 0.6 mg/ml (6B) borax concentration experimental subgroups**. The black arrows indicated SCEs occurred among human chromosomes of these subgroups. The sister chromatid exchange occurrence increased in the borax concentrations of 0.15, 0.2, 0.3 and 0.6 mg/ml experimental subgroups comparing with the control group.

Damage to genetic materials can be cytologically observed as SCE. The induction of SCE suggests exposure to genotoxins and possibly carcinogens. The increasing of SCE frequencies (mean SCE ± SD) when compared between the 0.1 mg/ml borax concentration experimental subgroup and the control group was not significant (*P *> 0.05). The increasing of SCE frequencies (mean SCE ± SD) when compared between the 0.15 mg/ml borax concentration experimental subgroup and the control group was significant (*P *< 0.05). This study also found the significant increases in the SCE frequencies (mean SCE ± SD) of human chromosomes in each of the three experimental subgroups (the borax concentration of 0.2, 0.3 and 0.6 mg/ml experimental subgroups) when compared to the control group (*P *< 0.05) (Table 1). The mean SCE ± SD in the experimental subgroups was increased when the borax concentration increased. The borax concentrations of 0.15, 0.2, 0.3 and 0.6 mg/ml significantly induced sister chromatid exchange in human chromosomes (*P *< 0.05) (Table 1). The borax concentrations of 0.15, 0.2, 0.3 and 0.6 mg/ml had the genotoxic effect to human chromosomes. When the human chromosomes exposed to borax, SCE occurrence in human chromosomes was increased in the borax concentrations of 0.15, 0.2, 0.3 and 0.6 mg/ml experimental subgroups comparing with the control group. Figure 5 showed the sister chromatid exchange occurrence in human chromosomes of the control group. Figures 6A, B showed the sister chromatid exchange occurrence in human chromosomes of the 0.15 and 0.6 mg/ml borax concentration experimental subgroups.

**Table 1:** Comparison of SCE frequencies in the control group and the experimental group

**Group**	**Mean SCE ± SD**	***P*-value**
The control group	0.74 ± 0.07	
The experimental group		
Borax 0.1 mg/ml experimental subgroup	2.20 ± 0.10	> 0.05*
Borax 0.15 mg/ml experimental subgroup	2.63 ± 0.11	< 0.05 *
Borax 0.2 mg/ml experimental subgroup	3.17 ± 0.14	< 0.05 *
Borax 0.3 mg/ml experimental subgroup	3.69 ± 0.18	< 0.05 *
Borax 0.6 mg/ml experimental subgroup	5.31 ± 0.19	< 0.05 *

* The statistical analysis comparing between the control group and each experimental subgroup.

## Discussion

Borax is the chemical substance which is toxic to human and animal. In human, borax is toxic to cells and has a slow excretion rate through the kidney. Kidney toxicity is the greatest, with liver fatty degenerations, cerebral edema and gastroenteritis. In animal, the testicular effects of borax were observed in rat, mouse and dog [14]. In rats, a single dose of 175 mg borax/kg bw was found to cause reversible disruption of tubular spermiation [15]. There were the reports about borax toxicity that it caused testicular atrophy, degeneration of seminiferous tubules, reduced sperm count, reduction in fertility in rats [16] and reduced fertility [17]. For the developmental toxicity of borax, the foetal body weight was decreased [18], minor skeleton variation with the exception of short rib XIII in rats [19].

In this study, the results from MTT assay indicated that at the borax concentrations of 0.15, 0.2, 0.3 and 0.6 mg/ml, immune cell proliferation (lymphocyte proliferation) showed low proliferation as compared to that of control group (Figures 1, 2, 3, 4). The numbers of viable lymphocyte were decreased in lymphocyte culture treated with high dose of borax. The 0.6 mg/ml borax concentration had the most effectiveness to lymphocyte proliferation in this study. It was toxic for cellular proliferation. Borax effects lymphocyte proliferation and it may cause cytotoxicity in immune cell (lymphocyte).

SCE frequencies have been examined in many human diseases such as Bloom syndrome. A variety of chemical and physical agents exhibiting diverse modes of interaction with DNA. These agents are capable of inducing SCE. The SCE technique is a sensitive means of monitoring DNA damage. The borax concentrations of 0.15, 0.2, 0.3 and 0.6 mg/ml induced sister chromatid exchange in human chromosomes (*P *< 0.05) (Table 1, Figures 5, 6). The frequency of SCE was increased when the borax concentration increased. The SCE frequencies of human chromosomes increased significantly in the experimental group as compared with the control group, suggesting that borax may have genotoxic effect in human.

## Conclusion

Borax is used as a food additive in some countries. This study suggests that borax may effect on immune cell (lymphocyte) cytotoxicity and genetic damage. The consumer should be careful about eating the preserved food for their health.

## Competing interests

The author declares that they have no competing interests.
